# Stereospecificity Membrane Impact of Two Catechins on Red Blood Cells

**DOI:** 10.3390/antiox15030328

**Published:** 2026-03-05

**Authors:** Stefano Putaggio, Marco D’Alì, Annamaria Russo, Giuseppe T. Patanè, Daniele Caruso, Salvatore V. Giofrè, Ester Tellone, Nunzio Iraci

**Affiliations:** 1 Department of Chemical, Biological, Pharmaceutical and Environmental Sciences, University of Messina, Viale Ferdinando Stagno d’Alcontres 31, 98166 Messina, Italy; stefano.putaggio@studenti.unime.it (S.P.); marco.dali@studenti.unime.it (M.D.); giuseppe.patane@studenti.unime.it (G.T.P.); salvatorevincenzo.giofre@unime.it (S.V.G.); nunzio.iraci@unime.it (N.I.); 2 “Prof. Antonio Imbesi” Foundation, University of Messina, 98100 Messina, Italy; 3 Complex Operational Unit, Clinical Pathology, Papardo Hospital, 98158 Messina, Italy; caruso.daniele1985@libero.it

**Keywords:** flavonoids, erythrocytes, Band 3 protein, anion flux kinetics, oxidative state, in silico studies

## Abstract

Catechins are characterized by a basic structure consisting of two benzene rings and a hydropyran heterocyclic ring. In (-)-epicatechin (ECT), the substituents in C2 and C3 of the dihydropyran ring are in *cis* conformation, whereas in (+)-catechin (CT), they are in *trans* conformation. Catechins tend to interact with membrane proteins, affecting their activity and/or function and metabolic processes. In this study, the impact of CT and ECT on erythrocyte membrane and cell functions was analyzed. Surprisingly, although the two compounds have a very similar structure that differs only in the orientation of the hydroxyl group in C3, they promote different effects on anion exchange through the phospholipid bilayer and on the release of ATP from cells. Anion transport mediated by Band 3 protein is reduced in the presence of CT compared with ECT which conversely increases it, and this observation aligns with the mechanisms of action we hypothesized in silico for the two compounds. Finally, ECT causes an increase in intracellular ATP levels unlike CT, and both molecules cause a decrease in ATP released from the erythrocyte. These findings could pave the way for further studies aimed at better understanding of the potential properties and structure–activity relationships of these molecules.

## 1. Introduction

**Figure 1:**
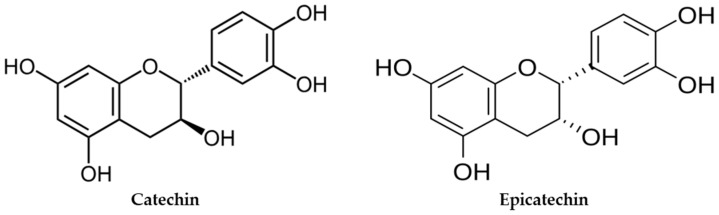
Structure of catechin (CT) and epicatechin (ECT).

Catechins are flavanols, a subgroup of flavonoids found in many foods of plant origin, in green tea, or even in cocoa beans (*Camellia sinensis and Theobroma cacao*) [1]. Catechins offer numerous health benefits, with antioxidant, anti-inflammatory, and neuroprotective properties, and are involved in the management of numerous diseases, such as diabetes, cardiovascular disease, metabolic syndrome, and a reduction in cholesterol and lipid oxidation [2,3,4,5,6,7]. There are some types of catechins characterized by two benzene rings (rich in -OH groups) and a heterocyclic dihydropyran ring carrying an -OH group on C3. The antioxidant capacity is mainly based on the arrangement of these hydroxyl groups and their ability to donate hydrogen atoms. In addition, on the heterocyclic dihydropyran ring, it is possible to distinguish two chiral centers at positions C2 and C3 that leads to four possible diastereoisomers. When the -OHs on C2 and C3 are oriented in the same direction, the configuration is often referred to as “*cis*” (2R, 3R) and the molecule is called epicatechin (ECT), whereas when OHs are oriented in the opposite direction specifically (2R,3S), it is called catechin (CT) (Figure 1) [8].

Studies have already been conducted on the effect of ECT on the metabolism of red blood cells (RBCs), demonstrating its protection against oxidant agents such as oxygen peroxide and confirming the beneficial action of catechins [9,10,11,12]. RBCs have a relatively simple structure compared to other cells. They lack a nucleus and organelles to make more room to store hemoglobin (Hb), the protein responsible for oxygen transport, and possess peculiar membrane characteristics that allow them high flexibility to deform through small blood vessels. The RBC’s membrane consists of a lipid bilayer and a protein network arranged under the inner surface, with both structures working in concert to maintain the shape of the cell, deformability, and function. Among the transmembrane proteins, anion exchanger 1 (AE1), also known as Band 3 or SLC4A1, is one of the main integral membrane proteins that catalyzes the electroneutral exchange of bicarbonate (${HCO}_{3}^{-}$) for chloride (Cl^−^) across the RBC membrane. AE1 functionality is essential for the efficient removal of carbon dioxide (CO_2_) from tissues to the lungs for exhalation. In intact RBCs, AE1 primarily exists in a stable dimer with some dimers that may associate to form tetramers. Each dimer subunit has a binding site for stilbene sulfonate, a known inhibitor of anion transport. This binding site is located on the outer membrane surface of AE1, and the binding of stilbene disulfonates does not directly prevent anion binding at the substrate site, but instead alters the protein’s conformation, leading to a reduction in anion exchange. Structurally, AE1 consists of 14 transmembrane (TM) segments for each monomer, with the N-terminal regions of TM3 and TM10 positioned opposite each other at the center of the protein, separated by a cavity that encompasses the anion binding site. The protein is arranged in a cytoplasmic domain (CD) comprising both N- and C-terminal regions, and a transmembrane domain with a core (TM1–4 and TM 8–11) and a gate domain (TM5–7 and TM12–14), with the anion-conducting pathway located between these domains [13]. CD serves as a crucial hub for various proteins, and it is responsible for building a multiprotein complex, anchoring AE1 with various cytoskeletal proteins including ankyrin, spectrin, protein 4.1, and protein 4.2 [14]. CD also competitively binds to deoxygenated hemoglobin and glycolytic enzymes like aldolase, glyceraldehyde-3-phosphate dehydrogenase, and phosphofructokinase [15,16]. Previous studies demonstrated the antioxidant effects of some flavonoids on RBCs; this article investigates the mechanisms underlying the modes and behaviors of catechin and epicatechin interactions with the erythrocyte membrane, specifically focusing the analysis on their interplay with AE1 and their influence on RBC metabolism. In fact, catechins, due to their hydrophobic nature, are drawn within the lipid bilayer where they partly accumulate, forming hydrogen bonds with lipid head groups [17].

## 2. Materials and Methods

### 2.1. Reagents and Compounds

All reagents were purchased from Sigma Aldrich (St. Louis, MO, USA). Human blood was collected from 20 healthy donors (10 males and 10 females) aged 27–30 years. All participants had a standard MDI, had not taken any medications in the 30 days prior to collection, and regularly followed a Mediterranean-style diet. Erythrocytes were stored in tubes with ethylenediaminetetraacetic acid (EDTA) as an anticoagulant and used fresh. The study was approved by a local ethics committee (prot. 71-23, 5 April 2023) in accordance with the Declaration of Helsinki. CT and ECT solutions were prepared by solubilizing the compounds in dimethyl sulfoxide (DMSO). In all experiments, the final concentration of DMSO was less than 0.1%.

### 2.2. Preparation of Erythrocytes

Blood samples were centrifuged (J2-HS centrifuge, Beckman, Harbor Boulevard, Fullerton, CA, USA) at 3000 rpm, for 5 min at 4 °C. The supernatant was discarded, and the erythrocytes were washed 3 times with a NaCl solution (0.9%) and then resuspended in buffer (2-[4-(2-hydroxyethyl) piperazin-1-yl] ethanesulfonic acid (HEPES)), 20 mOsmol, at pH 7.4. The osmolarity of the buffer was evaluated with Omostat OM-6020 (Daiichikagakuco, Kyoto, Japan). All experiments were conducted with a hematocrit of 4%.

### 2.3. Calculation of the Percentage of Hemolysis and Methemoglobin Value

Following the washes, RBCs (4% hematocrit) were treated with ECT and CT (50.0 μM) for 12 h, in Hepes buffer (25.0 mM), pH 7.4. Then, the samples were centrifuged (3000 rpm, 5 min, 4 °C) and the supernatant was used for the evaluation of hemolysis, while the pellet was used for the evaluation of methemoglobin. Percent hemolysis was assessed by spectrophotometric analysis, using a Beckman DU 640 spectrophotometer (Harbor Boulevard, Fullerton, CA, USA), at 576 nm. The percentage of hemolysis was evaluated by the following formula:

$$H\left(\%\right)=\frac{Abs_{sample}-Abs_{0}}{Abs_{100}-Abs_{0}}$$

where H(%) indicates the percentage of hemolysis; AbS_0_ indicates the absorbance of the control (RBCs); Abs_sample_ indicates the absorbance of the samples in the presence of molecules; AbS_100_ indicates the absorbance of the sample lysed with distilled H_2_O.

The pellets obtained were used to assess methemoglobin (Met-Hb) through spectrophotometric analysis (range 500–680 nm), as reported by Zijlstra et al. [18].

### 2.4. Caspase 3 Activity

Following washes, erythrocytes were diluted (4% hematocrit) in Hepes buffer, pH 7.4, and incubated with CT (50.0 μM), for 30 min at 37 °C. The positive control was RBCs incubated with tert-butyl-hydroperoxide (t-BuOOH) 100.0 μM. Then, the samples were centrifuged at 3000 rpm, 4 °C, for 5 min, the supernatant was discarded, and the pellet was diluted with Hepes buffer 100.0 mM, pH 7.4, and lysed by sonication. Finally, the samples were centrifuged, and the supernatant was filtered through Microcon YM 30 to concentrate caspase. Then after incubation for 1 h at 37 °C with AcDEVD-pNA (caspase 3 substrate), pNA release was evaluated by spectrophotometric analysis at 405 nm.

### 2.5. Determination of Anion Flux

The functional activity of AE1 was quantified using sulphate as a tracer ion. Sulfate transport was selected over chloride due to its significantly slower exchange kinetics, which facilitates high-resolution temporal monitoring. Furthermore, the negligible endogenous intracellular concentration of sulfate allows for a high signal-to-noise ratio, ensuring that measured internal sulfate levels are a direct reflection of transmembrane flux [19]. RBCs, 4% hematocrit, were incubated with ECT and CT (50.0 μM) in buffer (35.0 mM Na_2_SO_4_, 90.0 mM NaCl, 25.0 mM Hepes, and 1.5 mM MgCl_2_), at pH 7.4, at 25 °C. At different time intervals (5, 15, 30, 60, 90, and 120 min), 10.0 μM of 4-acetamide-40-isothiocyanostilbene-2,20-disulfonic acid (SITS) was added to the mixture to block transport. Afterwards, the samples were centrifuged at 4000 rpm for 10 min, and the pellets were washed with NaCl (0.9%). Then, the erythrocytes were treated with perchloric acid (4%) and lysed with H_2_O. Subsequently, the samples were centrifuged, and the supernatant was treated with glycerol and H_2_O (1:1, *v*/*v*), 4.0 M NaCl, 1.0 M HCl, and 1.23 M BaCl_2_ ⋅2H_2_O. Absorbance was assessed in the range of 350–425 nm by Beckman spectrophotometer DU 640 (Harbor Boulevard, Fullerton, CA, USA) [19]. The kinetic of transport was obtained by the equation:

$$c\left(t\right)=c\infty \times \left(1-e-kt\right)$$

c(t): sulfate concentration at time t; c∞: intracellular sulfate concentration at equilibrium; kt: flux constant of sulfate influx.

### 2.6. Intracellular and Extracellular ATP Levels

RBCs, 4% hematocrit, were treated with CT (50.0 μM) in buffer (35.0 mM Na_2_SO_4_, 90.0 mM NaCl, 25.0 mM Hepes, 1.5 mM MgCl_2_), pH 7.4, for 30 min at 37 °C. Then, trichloroacetic acid (TCA) (15%) was added to stop the reaction, and the samples were centrifuged at 3000 rpm for 5 min at 4 °C. The obtained supernatant is used for the evaluation of extracellular ATP concentration and the pellet for evaluation of intracellular ATP; the analysis was conducted with the Bio Orbit 1251 luminometer (Bio-Orbit Oy, Turku, Finland). Briefly, 10.0 μL of the obtained supernatants were diluted with H_2_O (1:100), and then diluted (1:1) with D-luciferin and Firefly Lantern (FLE 250) (Sigma-Aldrich, St. Louis, MO, USA) and read on the luminometer; the pellets were directly diluted (1:1) with a solution of D-luciferin and Firefly Lantern extract (FLE 250) and analyzed on the luminometer [20].

### 2.7. In Silico Studies

#### 2.7.1. Experimental 3D Structures Selection

The human AE1 sequence (accession code: P02730) was downloaded from UniProt [21] and used as a query in BLAST (version 2.17.0) [22] to identify high homology experimentally resolved structures. Six cryo-EM structures, showing complete identity and no gaps (Table 1), in complex with inhibitors such as H_2_DIDS (PDB ID: 4YZF), DIDS (PDB IDs: 7TY6 and 8T6V), niflumic acid (PDB ID: 7TY8), and dipyridamole (PDB ID: 8T6U) [23,24], or bicarbonate (PDB ID: 7TY7) [24], were selected and downloaded from the RCSB Protein Data Bank [25].

#### 2.7.2. AE1 Models Preparation

Each PDB structure was processed by retaining just a single monomer, including the anion transport channel, while the other subunit was removed, as the two subunits have been reported to operate independently [26,27]. For AE1 in complex with H_2_DIDS (PDB ID: 4YZF) [23], only the transmembrane region of the monomer was retained, and the two bound Fab fragments were removed. All the ligands were likewise removed from their respective structures. These models were then submitted to the Protein Preparation utility [28] to optimize the H-bond network and to relax the system by a short, restrained minimization that was stopped when the convergence criterion (0.30 Å RMSD for heavy atoms) was reached.

#### 2.7.3. Preparation of Ligands

ECT and CT were sketched using the Maestro GUI [29] and then submitted to the LigPrep utility [30] to generate low-energy 3D structures from each input ligand and account for possible tautomeric and ionization states at a pH range of 5−9. A single state, which was advanced to the following studies, was generated for CT and ECT.

#### 2.7.4. ${HCO}_{3}^{-}$ Binding Site Analysis

The analysis of the ${HCO}_{3}^{-}$ binding site in the experimental Cryo-EM structure (PDB ID: 7TY7) was performed using SiteMap [31]. Default settings were used and the binding site was evaluated within 6 Å from the bound ${HCO}_{3}^{-}$ atoms. The site was characterized by a SiteScore of 1.13, an exposure value of 0.52, an enclosure value of 0.89, and a volume of 272,685 Å^3^.

#### 2.7.5. Docking Grids Generation

The prepared AE1 models were aligned to the reference structure 7TY7 using the Schrödinger Maestro interface [29], and the docking grids were centered on the centroid of the Sitemap-defined site points (x: 8.98, y: 4.12, z: 43.23). The docking space was set as an 8000 Å^3^ cubic box, and the diameter midpoint of each docked ligand was required to stay within a smaller, nested 3375 Å^3^ cubic box. No scaling was applied for the van der Waals radii of nonpolar receptor atoms.

#### 2.7.6. ECT and CT Docking Simulations

After grids preparation, ECT and CT were docked using a previously reported stepwise protocol [32,33], considering only the inhibitor-bound structures (i.e., 4YZF, 7TY6, 7TY8, 8T6U, and 8T6V) as docking targets. Ligands were first flexibly docked using Glide SP [34,35], retrieving a maximum of five poses per compound. To avoid redundancy, poses with RMSD or maximum atomic deviations below 2.0 Å were discarded as duplicates. Each distinct docking pose was then refined and rescored with Glide XP [36]. Refined docking poses were finally ranked by docking score, retaining only the top-scoring result for each ligand (Table 2). Ligand nonpolar atom vdW radii was scaled by a factor of 0.80.

#### 2.7.7. ${HCO}_{3}^{-}$ Docking

To assess the effect of ECT presence on ${HCO}_{3}^{-}$ binding affinity, the anion was docked using the docking-predicted ECT/AE1 complex (based on the 4YZF experimental structure—see Material and Methods and Table 2) and the apoform of 4YZF. Two cubic Glide docking grids generated starting from the above-mentioned models, centered on the centroid of the experimental ${HCO}_{3}^{-}$ bound conformation (PDB ID: 7TY7), and their volume was set to 1000 Å^3^, requiring the diameter midpoint of each docking ligand to stay within a smaller, nested 125 Å^3^ cubic inner box. No scaling was applied for the van der Waals radii of nonpolar receptor atoms. ${HCO}_{3}^{-}$ was first flexibly docked against the two grids using Glide SP, retrieving five poses per compound. To avoid redundancy, poses with an RMSD below 1.5 Å, or maximum atomic deviation below 2.0 Å were discarded as duplicates. Each pose was then rescored with Glide XP, and the best-scoring bound conformation for each grid was selected. The obtained XP scores for ${HCO}_{3}^{-}$ were −4.406 and −2.398, in the presence and in the absence of ECT, respectively.

#### 2.7.8. Molecular Dynamics (MD) Simulations

The best-scoring predicted bound conformations of the ECT/${HCO}_{3}^{-}$/AE1 and CT/AE1 complexes (derived from 4YZF) were submitted to Molecular Dynamics simulations. MD-simulated environments were set up using Desmond [37,38]. The complexes were inserted into an explicit POPC bilayer, based on the coordinates of 8T6U entry from the OPM database [39]. Solvation was treated explicitly using the TIP3P water model [40]. The systems were neutralized by Na^+^ and Cl^−^, which were added to a final concentration of 0.15 M. Prior to MD production stage, membrane/ligand/protein systems were equilibrated by means of the standard equilibration stepwise protocol for membrane proteins distributed with Desmond. After system equilibration, 240 ns-long MD simulations were carried out at 300 K in the NpγT ensemble, using a Nose–Hoover chain thermostat and a Martyna–Tobias–Klein barostat. Backbone heavy atoms were constrained during the production stage (1 kcal/mol). MD trajectories were analyzed using the Simulation Interaction Diagram embedded in the Schrodinger Suite. The OPLS2005 [41] force field was used in all simulations.

### 2.8. Statistical Analysis

Data were expressed as the means of three different experiments (technical repeats) ± standard deviation (SD) and were statistically evaluated using one-factor analysis of variance (ANOVA), followed by the Tukey–Kramer test (GraphPad Software, version 8.0, San Diego, CA, USA). Values of *p* obtained less than or equal to 0.05 were considered significant.

## 3. Results

### 3.1. Effect of Catechin and Epicatechin on Erythrocyte Membrane

**Figure 2:**
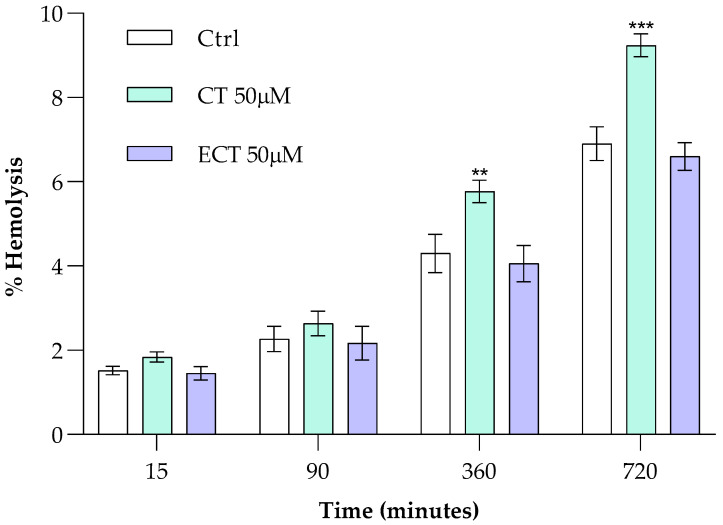
Effect of CT and ECT on the erythrocyte membrane, after 15, 90, 360, and 720 min of incubation time. Values are the mean ± SD of at least three different experiments. ** *p* < 0.01 vs. ctrl, *** *p* < 0.001 vs. ctrl. Statistical analysis performed by one-way ANOVA followed by Tukey’s test.

Catechins, due to their hydrophobic nature, tend to accumulate within cell membranes, sometimes causing slight expansion and affecting membrane properties. This interaction can alter membrane fluidity, permeability, and organization, and so the first step in our study was to evaluate the impact of ECT and CT on the integrity of the lipid bilayer through estimation of hemolysis percentage. By measuring the amount of hemoglobin released from RBCs, the results show that CT (50.0 μM) and ECT (50.0 μM) do not cause significant damage to the erythrocyte after a few minutes of treatment, up to a time of 90 min, compared to the control (Figure 2). However, prolonged incubation (360 to 720 min) causes a reverse trend for the effect of CT on membrane integrity. Although the protective effect of ECT is observable at all incubation times tested, CT appears to alter the erythrocyte membrane by increasing its vulnerability to lysis.

### 3.2. Effect of the Two Stereoisomers on the Health State of Red Blood Cells

**Figure 3:**
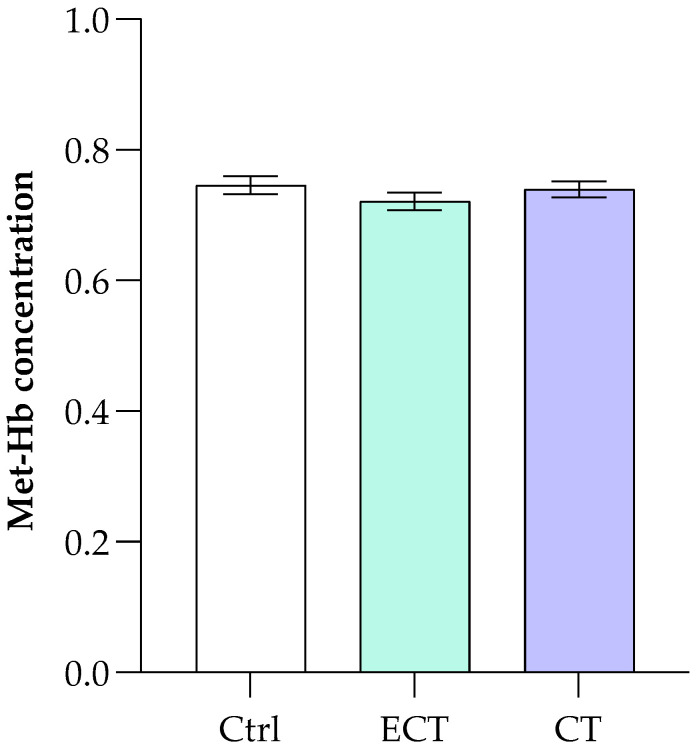
Effect of CT and ECT on the Met-Hb concentration measured at the end of the incubation time (720 min) of erythrocytes. The results are compared with erythrocytes (ctrl) treated under the same experimental conditions but in the absence of CT and ECT (50.0 μM). Values are the mean ± SD of at least three different experiments. Statistical analysis performed by one-way ANOVA followed by Tukey’s test.

Many molecules can act on hemoglobin by modifying its functionality and/or oxidizing it by inducing the formation of methemoglobin (Met-Hb), a condition in which the oxidation of ferrous ion to the ferric state (Fe^3+^) reduces the Hb’s ability to bind and release oxygen. In this view, the influence of CT and ECT was assessed on the oxidative state of Hb to evaluate their impact on protein function. In line with the existing scientific literature, the results in Figure 3 show no significant change in Met-Hb levels in RBCs after incubation for 12 h with CT and ECT (50.0 μM) compared with control.

### 3.3. Influence of Catechin and Epicatechin on AE1 Functionality

**Figure 4:**
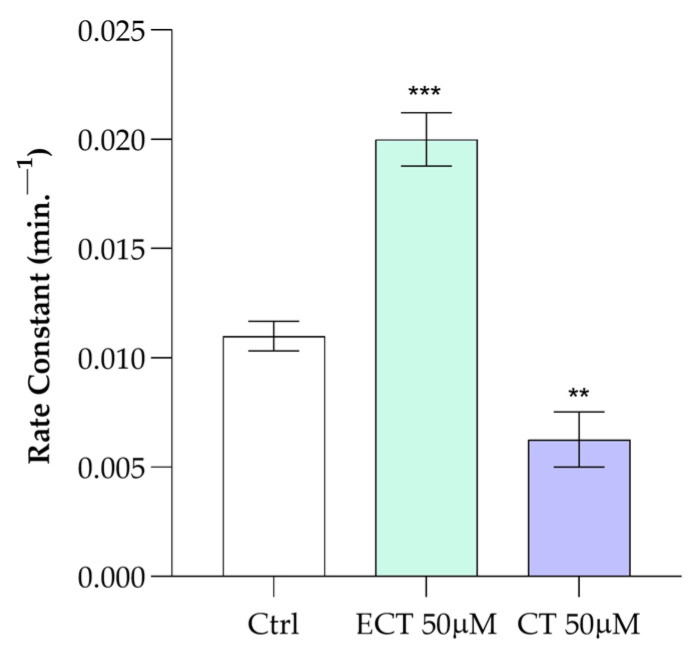
Rate of anion exchange in normal human erythrocytes, at 25 °C, treated with ECT and CT (50.0 μM) compared with RBCs (ctrl) under the same experimental conditions, in the absence of CT and ECT. Values are the mean ± SD of at least three different experiments. ** *p* < 0.01 vs. ctrl, *** *p* < 0.001 vs. ctrl. Statistical analysis performed by one-way ANOVA followed by Tukey’s test.

To study the impact of the two molecules on membrane structure, the AE1 efficiency of ${HCO}_{3}^{-}$/Cl^−^ exchange was tested in the presence of ECT and CT (50.0 μM). The results in Figure 4 show significant differences in the anion transport rate of AE1. In detail, it was observed that the presence of CT leads to a reduction in anion flux of about 40% compared to control. ECT, on the other hand, results in a significant increase in anion flux of about 80% compared to control.

Multiple factors can modulate AE1 activity and influence its anion exchange efficiency. While the primary regulatory site is the cytoplasmic domain of the protein, conformational changes in the transmembrane domain, the direct site of Cl^−^/${HCO}_{3}^{-}$ exchange induced by CT and ECT, could also affect transport efficiency through modifications of membrane fluidity and changes in the accessibility of the transport site [42,43,44]. In addition, the presence of the two molecules could trigger phosphorylation or oxidative events that further contribute to the regulation of AE1 activity.

### 3.4. Influence of CT on ATP Levels

**Figure 5:**
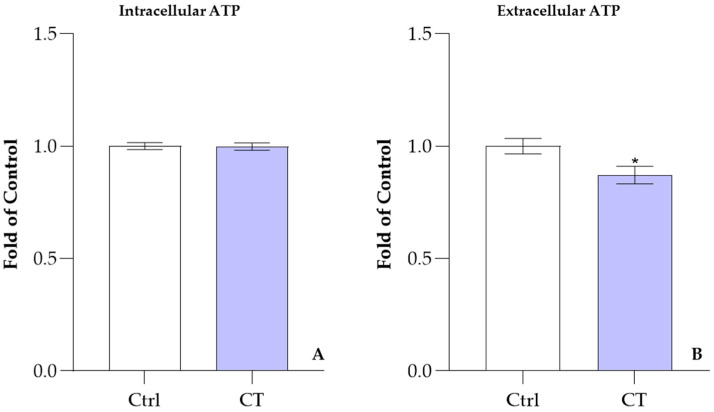
Changes in ATP concentration in the presence and absence of CT 50.0 μM compared to control (ctrl), after 30 min of incubation. Section (**A**) and section (**B**) show intracellular and extracellular ATP levels compared to control (ctrl). The normalized values with respect to the control (1) are the average ± SD of at least three different experiments. * *p* < 0.05 vs. ctrl. Statistical analysis performed by one-way ANOVA followed by Tukey’s test.

Another membrane-associated process that may be influenced by the presence of CT and ECT within the lipid bilayer is ATP release from RBCs. To test this hypothesis, ATP release was measured in RBCs. As shown in Figure 5, after 30 min of incubation with 50.0 μM CT, intracellular ATP levels remain largely unchanged (section A), whereas extracellular ATP release is reduced by approximately 15% (section B). Under the same experimental conditions, ECT induces a slight increase in intracellular ATP levels and a pronounced reduction in ATP release compared to CT, consistent with the effects reported in our previous publication [9] and confirming the different actions exerted by the two isomers on the erythrocyte membrane and, more generally, on RBCs.

### 3.5. Influence of CT on Caspase 3 Activity

**Figure 6:**
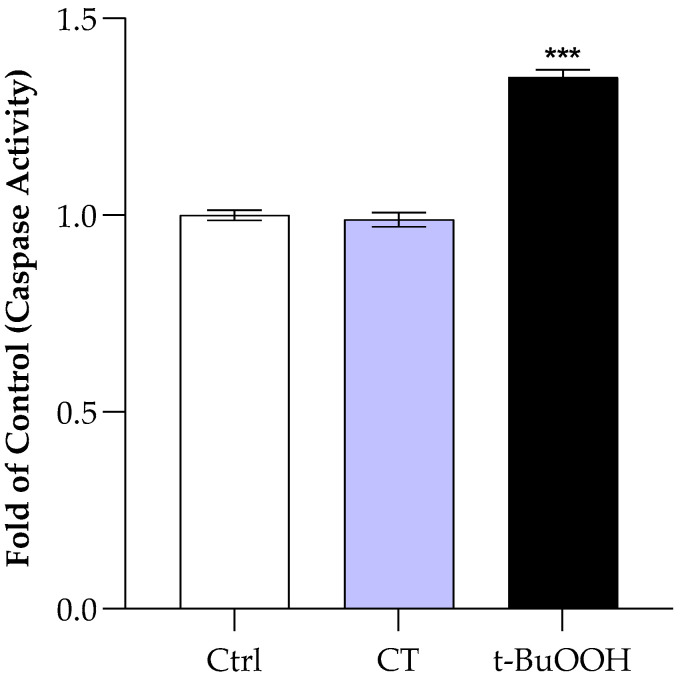
Caspase 3 activity measured in RBCs in the absence (ctrl), treated with CT (50.0 μM), or with t-BuOOH 100.0 μM after 30 min of incubation. Values are the mean ± SD of at least three different experiments. *** *p* < 0.001 vs. ctrl. Statistical analysis performed by one-way ANOVA followed by Tukey’s test.

The decrease in anion transport drives the cell towards abnormal CO_2_ detoxification. The slowdown of AE1 could cause cytosolic acidification, which in turn could affect the activation of caspase 3, the first step of the apoptotic process. To evaluate this hypothesis, caspase 3 activity was measured on RBCs after treatment for 30 min with CT 50.0 μM. The results visible in Figure 6 have been compared with those obtained in RBCs, under the same experimental conditions, incubated in the absence and in the presence of a known oxidizing agent, t-BuOOH [45]. The analysis does not show caspase activation and the results obtained are in line with what is already present in the literature on the effect of ECT [9]. In fact, even in this case, there was no enzymatic activation in the presence of CT.

The absence of caspase 3 activation in CT-treated erythrocytes suggests that the observed inhibition of AE1 may be linked to a direct interaction with CT. Typically, caspase 3 mediates the cleavage of the N-terminal cytoplasmic domain of AE1, which disrupts the membrane–cytoskeleton connection and reduces anion flux [42]. From this perspective, the increase in hemolysis after 720 min of exposure to CT could be explained by a direct biophysical interaction between CT and the cell membrane, rather than by the trigger of a metabolic pathway involving caspase 3 activation.

### 3.6. In Silico Studies

**Figure 7:**
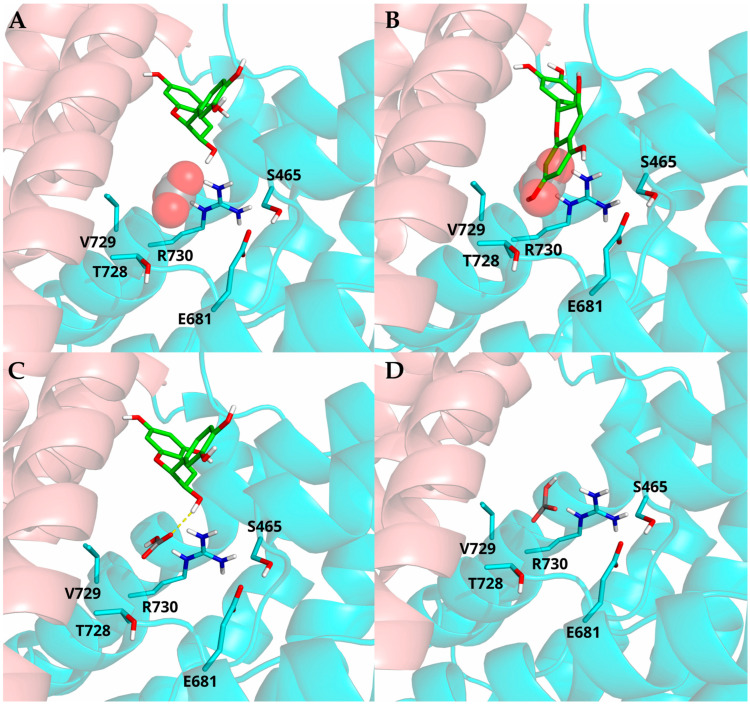
In silico analysis of ECT and CT binding to AE1. (**A**,**B**) Docking-predicted bound conformations of ECT (**A**) and CT (**B**), depicted in green sticks. Experimental bound conformation of ${HCO}_{3}^{-}$ is represented by transparent spheres. (**C**) Docking-predicted bound conformations of ${HCO}_{3}^{-}$ (gray sticks) in the presence of ECT (green sticks). H-bond is represented by a yellow dash line. (**D**) Docking-predicted bound conformation of ${HCO}_{3}^{-}$, depicted in gray sticks. In all panels, core domain residues are represented by cyan cartoons and sticks, and the gate domain by salmon cartoons.

Our experimental results demonstrated that ECT and CT exert opposite effects on the bicarbonate and chloride (${HCO}_{3}^{-}$/Cl^−^) exchange mediated by AE1 in RBCs, with ECT-enhancing and CT-reducing anion flux. To investigate the potential molecular mechanisms underlying these effects, molecular mechanics simulations were performed using five Cryo-EM structures of human AE1 in complex with various inhibitors (see Materials and Methods). In all these structures, AE1 adopts an outward-facing conformation, exposing the anion binding site toward the extracellular side. Docking simulations were performed to predict the conformations of the CT/AE1 and ECT/AE1 complexes and among the different AE1 protein structures analyzed, the 4YZF target structure yielded the most favorable docking scores for both CT and ECT (Table 2—see Materials and Methods). ECT and CT exhibited comparable docking scores, occupying a site located at the interface between the core and gate domains, in proximity to the ion-conducting pore, similar to what was observed for the other inhibitors reported in the literature [23,24], but showing distinct binding modes. Specifically, ECT adopted a folded conformation that does not occlude the ${HCO}_{3}^{-}$/Cl^−^ binding site, thereby keeping the site accessible to the anions. The ligand binding site is lined by residues F464, S465, G466, T728, V729, and R730, together with E681, which may orient toward the pocket, as supported by literature reports (Figure 7A) [46]. In contrast, CT adopted an extended conformation spanning the two domains, with its 5,7-dihydroxyphenyl moiety occupying the substrate-binding site (Figure 7B), potentially preventing the access of both ${HCO}_{3}^{-}$ and Cl^−^, and thereby acting similarly to dipyridamole [24]. This mechanism is consistent with experimental results showing a CT-dependent reduction in ${HCO}_{3}^{-}$/Cl^−^ exchange.

Subsequently, we evaluated whether ECT may interact with ${HCO}_{3}^{-}$, potentially influencing the molecular recognition of the anion. To test this hypothesis, docking simulations of ${HCO}_{3}^{-}$ were performed on 4YZF, both in the presence and absence of ECT, to assess its effect on ${HCO}_{3}^{-}$ binding affinity. Docking results revealed that, in the presence of ECT, ${HCO}_{3}^{-}$ docked within its binding site with higher affinity (docking score: −4.406), forming a H-bond involving the hydroxyl group at C3 of ECT (Figure 7C). In contrast, in the absence of ECT, the ion exhibited reduced binding affinity (docking score: −2.398) (Figure 7D). The interaction observed between ECT and ${HCO}_{3}^{-}$ may account for the more favorable docking score of ${HCO}_{3}^{-}$ and could also contribute to enhanced ${HCO}_{3}^{-}$ stability within its binding site, potentially facilitating its transport through the protein and ultimately contributing to the observed increase in anion flux.

**Figure 8:**
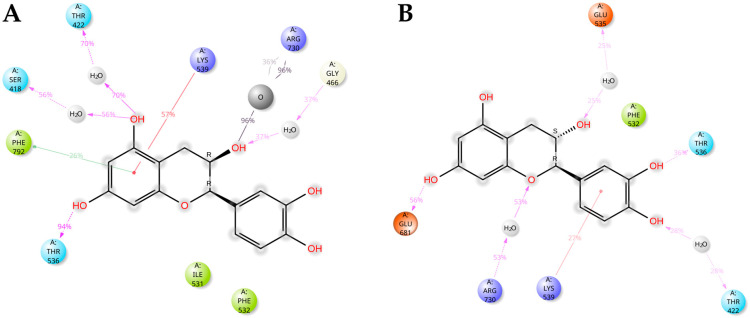
Molecular dynamics simulations of ECT/AE1 and CT/AE1 complexes. (**A**,**B**) Ligand interaction diagrams of 240 ns-long MD simulations of AE1 in complex with ECT (**A**) and CT (**B**). Only residues interacting with the ligand for at least 60 ns out of 240 ns of MD simulation time are shown. Residues are colored according to the following scheme: cyan, polar; green, hydrophobic; purple, charged (positive); red, charged (negative); light gray, water molecule; dark gray, ${HCO}_{3}^{-}$ molecule (identified by O); white, glycine. Gray halos highlight solvent exposure. H-bonds are represented by magenta arrows (dashed when side chain atoms are involved); the interaction between R730 and ${HCO}_{3}^{-}$ exhibits both ionic and H-bonding character (gray lines). Green solid lines represent π-π interactions, while red solid lines represent π-cation interactions.

Docking-predicted CT/AE1 and ECT/${HCO}_{3}^{-}$/AE1 models were challenged, in terms of conformation stability, by MD simulations that supported the stability of complexes and the proposed mechanisms of action, as both complexes remained stable during the simulation (Figure S1—Supplementary Materials). In particular, during the simulation, ECT maintained the docking-predicted H-bond with ${HCO}_{3}^{-}$, mediated by the hydroxyl group at C3. ${HCO}_{3}^{-}$, in turn, was found to interact with several residues, forming both ionic and H-bonding interactions with R730, the key residue directly implicated in anion transport [24]. Additionally, ECT formed direct H-bonds with T536, water-mediated H-bonds with S418, T422, and G466, hydrophobic interactions involving I531 and F532, as well as π–π stacking with F792 and a π–cation interaction with K539, with all these interactions collectively contributing to the stabilization of ECT within the binding site (Figure 8A). MD simulation of CT confirmed the stable occupation, by means of its 5,7-dihydroxyphenyl moiety of the substrate-binding site, thereby hindering the anions access. CT forms direct H-bonds with T536 and E681, as well as water-mediated H-bonds with T422 and R730, hydrophobic contacts with F532, and a π–cation interaction with K539, which altogether contribute to the stabilization of CT within the binding site (Figure 8B).

Therefore, the in silico analyses overall suggest a rationale for how the distinct configurations of ECT (*cis* configuration) and CT (*trans* configuration), particularly the orientation of the hydroxyl group at C3, may differentially modulate AE1-mediated anion transport, with ECT increasing and CT reducing anion flux, although experimental confirmation of this mechanism would require orthogonal validation through biochemical and functional assays (e.g., inhibitor competition, mutagenesis, and quantitative binding/transport measurements).

## 4. Discussion

The antioxidant effect of CT has been well demonstrated in various systems both in vitro and in vivo [47,48,49]. The effects of CT structure have been largely explained by their metal-chelating properties and their different interactions with functional proteins, but it is conceivable that membrane lipids can recognize the slight structural differences in these bioactive molecules and show specific responses. In this context, we have tried to give an answer to the different actions exerted by CT and ECT on RBCs membrane. In fact, the differences in the orientation of the C2 and C3 substituents seem to be the cause of the different modes of action of the two stereoisomers against important processes that take place at the membrane level, such as anion exchange and ATP release from the cell. Both have been shown to be acquired by RBCs and accumulate at the membrane level; however, the way stereoisomers interact with the bilayer appears to be different [10]. Tsuchiya et al. had already shown that the accumulation and interaction of catechins led to physical changes and a more rigid membrane structure; specifically, due to stereospecific activity, ECT in *cis* form is more efficient in reducing membrane fluidity than CT in *trans* conformation [50]. From a functional point of view, the different modes of interaction of the two stereoisomers with AE1 seems to correlate well with the results related to the different hemolytic fragility. In detail, the stimulating effect of ECT on AE1 anion exchange could be due to the direct action of ECT with the outer RBC membrane. In this perspective, the accumulation of ECT on the bilayer would seem to cause conformational changes in the channel protein favoring an “open” structure, making it more available for the passage of anions. This event can be interpreted as advantageous because a faster anion exchange favors the elimination of CO_2_ by reducing the possibility of generating ROS that could contribute to membrane disaggregation. This hypothesis is supported by the protective effect detected against the fragility of the membrane even after prolonged incubation times. ECT insertion into the lipid bilayer, stabilizing the membrane and decreasing its fluidity, combined with its inherent radical-scavenging ability, could also contribute to reducing free radical access within the bilayer, helping shield lipids and proteins from oxidative damage. This membrane localization further strengthens the antioxidant properties of the molecule, making it a powerful protector against oxidative damage [51]. By contrast, the effect of CT seems to be dangerous especially for long incubation times. In this case, CT interacting differently with AE1 inhibits the transmembrane anion flux. This causes a dangerous slowdown in the elimination of CO_2_ and peroxynitrite from the cytosol, leading to the generation of secondary toxic radicals including carbonate, nitrogen dioxide, and hydroxyl, and consequent oxidative damage to the RBCs [52,53]. The results on hemolysis in the presence of CT corroborate what has been hypothesized and are in line with literature [10]. In fact, CT, unlike ECT, does not protect the RBCs membrane, and prolonged incubation times increase the fragility of the membrane. In silico studies helped us investigate why the two compounds could modulate AE1–mediated anion exchange in RBCs in opposite ways. They suggest that ECT and CT might bind into the same binding pocket occupied by known AE1 inhibitors, yet their distinct binding modes lead to divergent functional outcomes. Indeed, simulations suggest that ECT enhances ${HCO}_{3}^{-}$ binding affinity, mainly through the formation of a H-bond (between its C3-OH and ${HCO}_{3}^{-}$ that stabilizes the anion within its binding site, potentially facilitating AE1/${HCO}_{3}^{-}$ molecular recognition, thereby contributing to the observed increase in anion flux. By contrast, CT occupies the anion binding site through its 5,7-dihydroxyphenyl moiety, thus hindering the anion’s access and potentially contributing to a reduction in ${HCO}_{3}^{-}$/Cl^−^ exchange. The hypothesis of a direct effect of the two molecules, following their accumulation in the lipid bilayer, is confirmed by the action detected on ATP release from the RBC. In this case, the effect of the stereoisomers is the same, both reducing the release of ATP but with a different intensity [9]. This response is attributable to an alteration in the fluidity of the membrane, because all the stereoisomers of catechins reduce the fluidity of the membrane by acting on the hydrophilic and hydrophobic regions of the bilayer [48]. In this context, the greater efficiency of ECT in reducing membrane fluidity compared to CT, demonstrated by Tsuchiya et al., may be related to the reduction in ATP release from RBCs. In fact, membrane stiffness (which is associated with aging or metabolic diseases such as diabetes) causes an alteration in normal cellular functions, including the release of ATP driven by mechanical changes in the shape of RBCs because of its journey into the bloodstream [50,54,55]. When the membrane is less fluid and more rigid, RBCs are less adept at deformation, leading to a significant reduction in ATP release. This condition affects tissue oxygenation; in fact, rigid RBCs will have difficulty adapting to different conditions along the blood stream. They will be less able to deform in response to mechanical stimuli, resulting in significantly lower release of ATP as a signal molecule to surrounding tissues to promote vasodilation, increased blood flow, and tissue oxygenation. In addition, reduced membrane fluidity impairs the activity and movements of proteins in the membrane, including ATPases responsible for maintaining cell’s ionic balance, cellular volume, and membrane structural integrity.

## 5. Conclusions

Herein, the presented results characterize the different interactions of the two stereoisomers CT and ECT with the erythrocyte membrane, with the AE1 protein in particular. The interaction patterns predicted by in silico studies give insight into CT- and ECT-mediated modulations of the AE1 anion exchange activity, which also highlights the different antioxidant potentials of the two catechins. Although both compounds bind to the same pocket on AE1, their distinct binding modes lead to divergent outcomes: ECT stabilizes ${\mathrm{HCO}}_{3}^{-}$ through the formation of a H-bond with the anion, potentially contributing to the observed increase in ${\mathrm{HCO}}_{3}^{-}$/Cl^−^ exchange, whereas CT occupies the anion binding site, obstructing ${\mathrm{HCO}}_{3}^{-}$ and Cl^−^ access, thus potentially contributing to the observed reduction in anion flux. Overall, a different impact of the two stereoisomers on RBC emerges, which, in accordance with the Tsuchiya results, also involves ATP release and tissue oxygenation [50]. In humans, the peak plasma concentration of CT and ECT after a moderate intake (e.g., a cup of green tea or a glass of red wine) typically ranges between 50 nM and 1 μM. Even with high-dose supplementation (800 mg), levels rarely exceed 2–5 μM. While in vitro studies often use higher concentrations (1–100 μM) than what is typically achieved in human plasma (sub-micromolar to low micromolar), they provide crucial insights into mechanisms, such as the inhibition of Low-Density Lipoprotein (LDL) oxidation and modulation of inflammatory pathways [56,57,58]. In this study, selected concentrations of 50–100 μM serve as a necessary “stress test” to identify fundamental stereoisomer binding modes that might otherwise remain obscured by the analytical noise inherent in lower-concentration studies. Moreover, this experimental approach made it possible to identify additional catechin-mediated effects on erythrocytes that may be physiologically relevant and contribute to their potential application as metabolic supplements.

## Figures and Tables

**Table 1:** BLAST alignment metrics for the selected PDB entries, retrieved using UniProt P02730 sequence as a query.

PDB IDs	E-Value	Score	Identity (%)	Positive (%)	Gaps (%)
4YZF	0	4459	100	100	0
7TY6	0	4459	100	100	0
7TY7	0	4459	100	100	0
7TY8	0	4459	100	100	0
8T6U	0	2529	100	100	0
8T6V	0	2529	100	100	0

**Table 2:** XP docking scores of ECT and CT.

Structure	XP Docking Score ECT	Structure	XP Docking Score CT
4YZF-H_2_DIDS	−7.416	4YZF-H_2_DIDS	−6.948
7TY6-DIDS	−7.184	8T6U-dipyridamole	−6.707
8T6U-dipyridamole	−6.901	7TY6-DIDS	−6.577
8T6V-DIDS	−6.307	8T6V-DIDS	−5.929
7TY8-NIF	−5.745	7TY8-NIF	−5.744

## Data Availability

The original contributions presented in this study are included in the article/Supplementary Materials. Further inquiries can be directed at the corresponding author(s).

## Supplementary Materials

The following supporting information can be downloaded at: https://www.mdpi.com/article/10.3390/antiox15030328/s1, Figure S1: RMSD of ECT and CT as a function of MD simulation time.

## References

[1] Musial C., Kuban-Jankowska A., Gorska-Ponikowska M. (2020). Beneficial Properties of Green Tea Catechins. Int. J. Mol. Sci..

[2] Patanè G.T., Putaggio S., Tellone E., Barreca D., Ficarra S., Maffei C., Calderaro A., Laganà G. (2023). Catechins and Proanthocyanidins Involvement in Metabolic Syndrome. Int. J. Mol. Sci..

[3] Yamagata K., Yamori Y. (2020). Inhibition of Endothelial Dysfunction by Dietary Flavonoids and Preventive Effects Against Cardiovascular Disease. J. Cardiovasc. Pharmacol..

[4] Barnaba C., Medina-Meza I.G. (2019). Flavonoids Ability to Disrupt Inflammation Mediated by Lipid and Cholesterol Oxidation. Adv. Exp. Med. Biol..

[5] Al-Ishaq R.K., Abotaleb M., Kubatka P., Kajo K., Busselberg D. (2019). Flavonoids and Their Anti-Diabetic Effects: Cellular Mechanisms and Effects to Improve Blood Sugar Levels. Biomolecules.

[6] Vargas F., Romecin P., Garcia-Guillen A.I., Wangesteen R., Vargas-Tendero P., Paredes M.D., Atucha N.M., García-Estañ J. (2018). Flavonoids in Kidney Health and Disease. Front. Physiol..

[7] Bernatova I. (2018). Biological activities of (−)-epicatechin and (−)-epicatechin-containing foods: Focus on cardiovascular and neuropsychological health. Biotechnol. Adv..

[8] Isemura M. (2019). Catechin in Human Health and Disease. Molecules.

[9] Russo A., Patanè G.T., Laganà G., Cirmi S., Ficarra S., Barreca D., Giunta E., Tellone E., Putaggio S. (2024). Epicatechin influences biochemical modification of human erythrocyte metabolism and membrane integrity. Int. J. Mol. Sci..

[10] Naparlo K., Bartosz G., Stefaniuk I., Cieniek B., Soszynski M., Sadowska-Bartosz I. (2020). Interaction of Catechins with Human Erythrocytes. Molecules.

[11] Maurya P.K., Rizvi S.I. (2009). Protective role of tea catechins on erythrocytes subjected to oxidative stress during human aging. Nat. Prod. Res..

[12] Colina J.R., Suwalsky M., Manrique-Moreno M., Petit K., Aguilar L.F., Jemiola-Rzeminska M., Strzalka K. (2019). Protective effect of epigallocatechin gallate on human erythrocytes. Colloids Surf. B Biointerfaces.

[13] Reithmeier A.F.R., Casey J.R., Kalli A.C., Sansom M.S.P., Alguel Y., Iwata S. (2016). Band 3, the human red cell chloride/bicarbonate anion exchanger (AE1, SLC4A1), in a structural context. Biochim. Biophys. Acta Biomembr..

[14] Jennings M.L. (2021). Cell physiology and molecular mechanism of anion transport by erythrocyte band 3/AE1. Am. J. Physiol. Cell Physiol..

[15] Bennett V., Stenbuck P.J. (1979). The membrane attachment protein for spectrin is associated with band 3 in human erythrocyte membranes. Nature.

[16] Low P.S. (1986). Structure and function of the cytoplasmic domain of band 3: Center of erythrocyte membrane-peripheral protein interactions. Biochim. Biophys. Acta Rev. Biomembr..

[17] Sirk T.W., Brown E.F., Sum A.K., Friedman M. (2008). Molecular dynamics study on the biophysical interactions of seven green tea catechins with lipid bilayers of cell membranes. J. Agric. Food Chem..

[18] Zijlstra W.G., Buursma A., Meeuwsen-van der Roest W.P. (1991). Absorption spectra of human fetal and adult oxyhemoglobin, de-oxyhemoglobin, carboxyhemoglobin, and methemoglobin. Clin. Chem..

[19] Romano L., Peritore D., Simone E., Sidoti A., Trischitta F., Romano P. (1998). Chloride-sulphate exchange chemically measured in human erythrocyte ghosts. Cell. Mol. Biol..

[20] Russo A., Patanè G.T., Putaggio S., Lombardo G.E., Ficarra S., Barreca D., Giunta E., Tellone E., Laganà G. (2024). Mechanisms Underlying the Effects of Chloroquine on Red Blood Cells Metabolism. Int. J. Mol. Sci..

[21] UniProt Consortium (2023). UniProt: The Universal Protein Knowledgebase in 2023. Nucleic Acids Res..

[22] Altschul S.F., Gish W., Miller W., Myers E.W., Lipman D.J. (1990). Basic local alignment search tool. J. Mol. Biol..

[23] Arakawa T., Kobayashi-Yurugi T., Alguel Y., Iwanari H., Hatae H., Iwata M., Abe Y., Hino T., Ikeda-Suno C., Kuma H. (2015). Crystal structure of the anion exchanger domain of human erythrocyte band 3. Science.

[24] Capper M.J., Yang S., Stone A.C., Vatansever S., Zilberg G., Mathiharan Y.K., Habib R., Hutchinson K., Zhao Y., Schlessinger A. (2023). Substrate binding and inhibition of the anion exchanger 1 transporter. Nat. Struct. Mol. Biol..

[25] Berman H.M., Westbrook J., Feng Z., Gilliland G., Bhat T.N., Weissig H., Shindyalov I.N., Bourne P.E. (2000). The Protein Data Bank. Nucleic Acids Res..

[26] Macara I.G., Cantley L.C. (1981). Interactions between transport inhibitors at the anion binding sites of the band 3 dimer. Biochemistry.

[27] Macara I.G., Cantley L.C. (1981). Mechanism of anion exchange across the red cell membrane by band 3: Interactions between stilbenedisulfonate and NAP-taurine binding sites. Biochemistry.

[28] Sastry G.M., Adzhigirey M., Day T., Annabhimoju R., Sherman W. (2013). Protein and ligand preparation: Parameters, protocols, and influence on virtual screening enrichments. J. Comput. Aided Mol. Des..

[29] Schrödinger, Inc. (2022). Maestro.

[30] Schrodinger, Inc. (2022). LigPrep.

[31] Schrödinger, Inc. (2022). SiteMap.

[32] Astolfi A., Iraci N., Sabatini S., Barreca M.L., Cecchetti V. (2015). p38α MAPK and Type I Inhibitors: Binding Site Analysis and Use of Target Ensembles in Virtual Screening. Molecules.

[33] Barreca M.L., Iraci N., Manfroni G., Gaetani R., Guercini C., Sabatini S., Tabarrini O., Cecchetti V. (2013). Accounting for Target Flexibility and Water Molecules by Docking to Ensembles of Target Structures: The HCV NS5B Palm Site I Inhibitors Case Study. J. Chem. Inf. Model..

[34] Friesner R.A., Banks J.L., Murphy R.B., Halgren T.A., Klicic J.J., Mainz D.T., Repasky M.P., Knoll E.H., Shelley M., Perry J.K. (2004). Glide: A New Approach for Rapid, Accurate Docking and Scoring. 1. Method and Assessment of Docking Accuracy. J. Med. Chem..

[35] Halgren T.A., Murphy R.B., Friesner R.A., Beard H.S., Frye L.L., Pollard W.T., Banks J.L. (2004). Glide: A New Approach for Rapid, Accurate Docking and Scoring. 2. Enrichment Factors in Database Screening. J. Med. Chem..

[36] Friesner R.A., Murphy R.B., Repasky M.P., Frye L.L., Greenwood J.R., Halgren T.A., Sanschagrin P.C., Mainz D.T. (2006). Extra Precision Glide: Docking and Scoring Incorporating a Model of Hydrophobic Enclosure for Protein–Ligand Complexes. J. Med. Chem..

[37] Bowers K.J., Chow D.E., Xu H., Dror R.O., Eastwood M.P., Gregersen B.A., Klepeis J.L., Kolossvary I., Moraes M.A., Sacerdoti F.D. Scalable algorithms for molecular dynamics simulations on commodity clusters. Proceedings of the 2006 ACM/IEEE Conference on Supercomputing (SC ’06).

[38] D.E. Shaw Research (2022). Desmond Molecular Dynamics System.

[39] Lomize M.A., Pogozheva I.D., Joo H., Mosberg H.I., Lomize A.L. (2012). OPM database and PPM web server: Resources for positioning of proteins in membranes. Nucleic Acids Res..

[40] Jorgensen W.L., Chandrasekhar J., Madura J.D., Impey R.W., Klein M.L. (1983). Comparison of simple potential functions for simulating liquid water. J. Chem. Phys..

[41] Jorgensen W.L., Maxwell D.S., Tirado-Rives J. (1996). Development and testing of the OPLS all-atom force field on conformational energetics and properties of organic liquids. J. Am. Chem. Soc..

[42] Roy S., Bayly C.I., Gareau Y., Houtzager V.M., Kargman S., Keen S.L., Rowland K., Seiden I.M., Thornberry N.A., Nicholson D.W. (2001). Maintenance of caspase-3 proenzyme dormancy by an intrinsic “safety catch” regulatory tripeptide. Proc. Natl. Acad. Sci. USA.

[43] Zhang D., Kiyatkin A., Bolin J.T., Low P.S. (2000). Crystallographic structure and functional interpretation of the cytoplasmic domain of erythrocyte membrane band 3. Blood.

[44] Campanella M.E., Chu H., Low P.S. (2005). Assembly and regulation of a glycolytic enzyme complex on the human erythrocyte membrane. Proc. Natl. Acad. Sci. USA.

[45] Saito M., Sakagami H., Fujisawa S. (2003). Cytotoxicity and apoptosis induction by butylated hydroxyanisole (BHA) and butylated hydroxytoluene (BHT). Anticancer Res..

[46] Su C.C., Zhang Z., Lyu M., Cui M., Yu E.W. (2024). Cryo-EM Structures of the Human Band 3 Transporter Indicate a Transport Mechanism Involving the Coupled Movement of Chloride and Bicarbonate Ions. PLoS Biol..

[47] Nain C.W., Mignolet E., Herent M.F., Quetin-Leclercq J., Debier C., Page M.M., Larondelle Y. (2022). The Catechins Profile of Green Tea Extracts Affects the Antioxidant Activity and Degradation of Catechins in DHA-Rich Oil. Antioxidants.

[48] Intra J., Kuo S.M. (2007). Physiological levels of tea catechins increase cellular lipid antioxidant activity of vitamin C and vitamin E in human intestinal Caco-2 cells. Chem. Biol. Interact..

[49] Yan Z., Zhong Y., Duan Y., Chen Q., Li F. (2020). Antioxidant mechanism of tea polyphenols and its impact on health benefits. Anim. Nutr..

[50] Tsuchiya H. (2001). Stereospecificity in membrane effects of catechins. Chem. Biol. Interact..

[51] Grzesik M., Naparło K., Bartosz G., Sadowska-Bartosz I. (2018). Antioxidant properties of catechins: Comparison with other antioxidants. Food Chem..

[52] Szabò C., Ischiropoulos H., Radi R. (2007). Peroxynitrite: Biochemistry, pathophysiology and development of therapeutics. Nat. Rev. Drug Discov..

[53] Tellone E., Ficarra S., Russo A., Bellocco E., Barreca D., Laganà G., Leuzzi U., Pirolli D., De Rosa M.C., Giardina B. (2012). Caffeine inhibits erythrocyte membrane derangement by antioxidant activity and by blocking caspase 3 activation. Biochimie.

[54] Forsyth A.M., Braunmüller S., Wan J., Franke T., Stone H.A. (2012). The effects of membrane cholesterol and simvastatin on red blood cell deformability and ATP release. Microvasc. Res..

[55] Racine M.L., Dinenno F.A. (2019). Reduced deformability contributes to impaired deoxygenation-induced ATP release from red blood cells of older adult humans. J. Physiol..

[56] Cai Z.Y., Li X.M., Liang J.P., Xiang L.P., Wang K.R., Shi Y.L., Yang R., Shi M., Ye J.H., Lu J.L. (2018). Bioavailability of Tea Catechins and Its Improvement. Molecules.

[57] Chow H.H., Hakim I.A. (2011). Pharmacokinetic and chemoprevention studies on tea in humans. Pharmacol. Res..

[58] Inami S., Takano M., Yamamoto M., Murakami D., Tajika K., Yodogawa K., Yokoyama S., Ohno N., Ohba T., Sano J. (2007). Tea catechin consumption reduces circulating oxidized low-density lipoprotein. Int. Heart J..

