# Supplementary figures and images for: Stereospecificity Membrane Impact of Two Catechins on Red Blood Cells
Source: Antioxidants (Basel). 2026 Mar 5;15(3):328. doi: 10.3390/antiox15030328 (PMC13024716; doi:10.3390/antiox15030328)

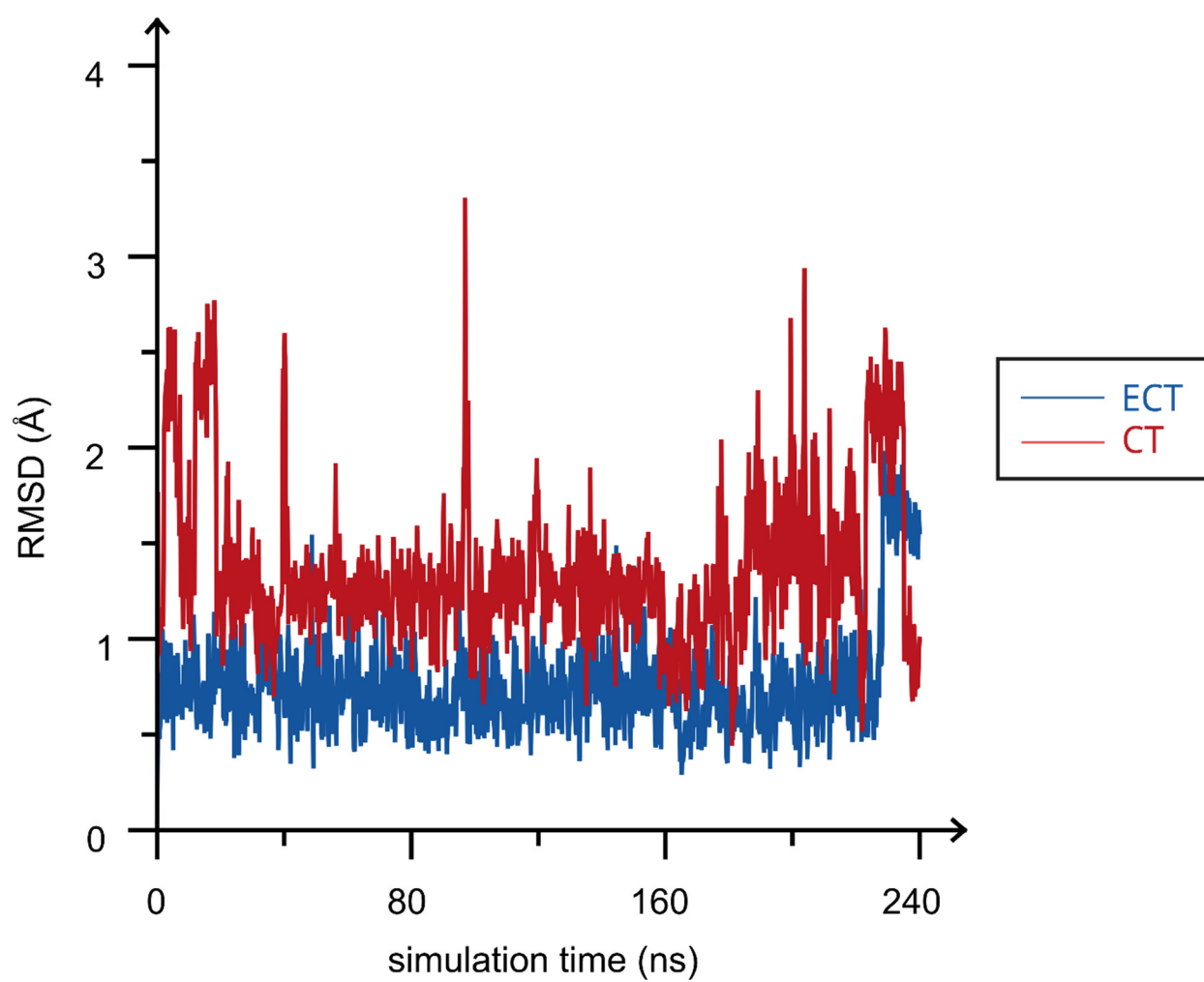

**Figure S1:** RMSD of ECT and CT as a function of MD simulation time.

Supplement: Supplementary file 1 [file antioxidants-15-00328-s001.zip › antioxidants-4173442-supplementary.pdf]
